# Connaissances des médecins généralistes de Mohammedia (Maroc) concernant le dépistage du cancer du sein

**DOI:** 10.11604/pamj.2016.24.243.9627

**Published:** 2016-07-15

**Authors:** Karima Zine, Samira Nani, Imad Ait Lahmadi, Abderrahmane Maaroufi

**Affiliations:** 1 Laboratoire d’Epidémiolgie, Faculté de Médecine et de Pharmacie de Casablanca, Maroc; 2 Centre Hospitalier Universitaire, Ibn Rochd de Casablanca, Maroc

**Keywords:** Cancer du sein, médecin généraliste, dépistage, Breast cancer, general practitioner, primary health care, screening

## Abstract

**Introduction:**

Le cancer du sein représente un problème de santé publique majeur au Maroc. C'est le premier cancer chez la femme. L'objectif de ce travail était d'évaluer les connaissances des médecins généralistes (MG) en matière de dépistage du cancer du sein dans la préfecture de Mohammedia Maroc.

**Méthodes:**

Nous avons mené une étude transversale, descriptive, exhaustive incluant les 97 MG exerçant dans les établissements de soins de santé de base du secteur public et privé de la province de Mohammedia.

**Résultats:**

Le taux de participation était de 87%. L'âge moyen des MG était de 49,6 ± 8,1. Quatre-vingt pour cent (n=55) des MG ont donné une incidence incorrecte, 77,6% (n=85) ont reconnu l'existence d'un plan national de prévention et de contrôle du cancer (PNPCC) au Maroc, et 67,1 des MG ont rapporté l'existence d'un registre du cancer au Maroc. Le secteur d'activité était associé significativement avec les connaissances des MG sur le PNPCC et sur l'existence d'un guide de détection précoce du cancer du sein avec respectivement (p=0,003 et p=0,001). Une association significative entre l'ancienneté et l'existence d'un guide de détection précoce du cancer du sein et d'un registre du cancer du sein a été retrouvée avec (respectivement p=0,005 et p=0.002).

**Conclusion:**

À la lumière de ces résultats il faudra renforcer les connaissances et les pratiques des MG par la promotion de la formation initiale et continue sur le dépistage.

## Abstract (en)

**Introduction:**

Breast cancer is a major public health problem in Morocco. It is the most common cancer in women. Our study aims to evaluate the extent of breast cancer awareness among general practitioners (GP) in the prefecture of Mohammedia, Morocco.

**Methods:**

We conducted a cross-sectional, descriptive, exhaustive study including 97 GP working in primary health care facilities (public and private sector) of the province of Mohammedia.

**Results:**

Participation rate was 87%. The average age of GP was 49.6 ± 8.1. Eighty percent (n = 55) of the GP misstated the incidence of breast cancer, 77.6% (n = 85) recognized the existence of a national plan to prevent and control cancer (NPPCC) in Morocco and 67.1% of GP reported the existence of a cancer registry in Morocco. General practice sector was significantly related to the awareness of NPPCC among GP and to the existence of guidelines for the early detection of breast cancer (p = 0.003 and p = 0.001 respectively). A significant relationship was found between seniority and the existence of guidelines for the early detection of breast cancer and a breast cancer registry (p = 0.005 and p = 0.002 respectively).

**Conclusion:**

In light of these results GP awareness and practices should be enhanced by promoting initial and continuing training on breast cancer screening.

## Introduction

Le cancer du sein est le premier cancer chez la femme dans le monde. Selon l’Organisation Mondiale de la Santé (OMS) 1,7 million des femmes ont un diagnostic de cancer du sein chaque année et en 2012, 6,3 millions de femmes vivaient avec un cancer du sein diagnostiqué au cours des cinq années précédentes. Depuis, les dernières estimations de 2008, l'incidence a augmenté de plus de 20%, et la mortalité de 14%. Le cancer du sein est la cause la plus fréquente de décès par cancer chez les femmes (522 000 décès) et le cancer le plus fréquemment diagnostiqué chez les femmes dans 140 des 184 pays couverts par GLOBOCAN dans le monde. Il représente maintenant un cancer sur quatre chez les Femmes [1]. L’American Cancer society (ACS) rapporte qu’en 2013 environ 232 340 nouveaux cas de cancer du sein ont été diagnostiqués et 39 620 femmes ont été mortes de cette maladie aux Etats Unis [2]. En France, 48 763 nouveaux cas ont été estimés en 2012 avec une mortalité de 11 886 décès [3]. En Afrique du Nord et au Moyen-Orient, le cancer du sein est également le premier cancer de la femme. Il représente 14 à 42 % de tous les cancers féminins avec une augmentation exponentielle [4]. Au Maroc, d’après les résultats du Registre des Cancers de la Région du grand Casablanca (RCRC) et du Registre des Cancers de Rabat (RCR) les incidences standardisées étaient respectivement de 39, 9 et de 49, 2 par 100.000 femmes (RCRC, 2012; RCR, 2012) [5]. Sa détection précoce permet d’instaurer une thérapeutique chirurgicale conservatrice moins lourde sur les plans psychologique et médicale, et permet d’améliorer le pronostic. Les moyens de dépistage sont basés sur la mammographie, l’examen clinique des seins (ECS) par un professionnel de santé et l’auto-examen des seins (AES) par les femmes elles-mêmes [6]. Ces deux dernières techniques simples et peu onéreuses semblent être plus réalistes et raisonnables comme méthodes de dépistage du cancer du sein dans les pays en voie de développement [7]. Des études ont montré que la mise en œuvre d’un programme de détection précoce, durant plusieurs années peut faire diminuer de 25 % le taux de mortalité due à cette maladie [7]. Depuis Mars 2010 le Maroc a lancé le Plan National de Prévention et de Contrôle du Cancer (PNPCC) qui a retenu parmi ses priorités la détection des cancers du sein et du col de l’utérus. Ce programme intéresse les femmes âgées entre 45 et 70 ans pour le cancer du sein. Les médecins généralistes (MG) sont les acteurs clés du dépistage, au-delà de leur place dans le système de soins et en raison de l’impact de leur discours auprès de leurs patientes, ils sont un des leviers de sensibilisation au dépistage [8]. En effet, plusieurs études ont montré que l’adhésion des femmes au dépistage du cancer du sein dépend d’une manière significative de l’attitude de leurs médecins [9–15]. Une politique nationale de dépistage de cancer du sein passe obligatoirement par une forte mobilisation du MG. Son niveau de connaissances, attitudes et pratiques concernant le cancer du sein et son dépistage sont des facteurs importants pour qu’il soit un acteur efficace dans tout programme de contrôle de cette maladie [16]. L’objectif de ce travail était d’évaluer les connaissances du MG concernant le cancer du sein et son dépistage dans la ville de Mohammedia au Maroc.

## Méthodes

**Type et population d’étude:** une étude transversale à visée descriptive a été menée chez les MG de la province de Mohammedia, au Maroc. La population totale de cette région a été estimée en 2012 à 367000 habitants. L’étude était exhaustive, les 97 MG exerçant dans les structures de première ligne de la province de Mohammedia ont été inclus: 49 appartenaient au secteur privé et 48 appartenaient au secteur public (Réseau de soin de santé de base (RSSB)).

**Variables à l’étude:** les variables étudiées incluaient les caractéristiques sociodémographiques et professionnelles des médecins (le genre, l’ancienneté d’exercice, le milieu d’exercice et le secteur d’activité), les connaissances concernant les outils stratégiques de lutte contre le cancer (l’existence d’un plan national de lutte contre le cancer (PNPCC), l’existence d’un guide de détection précoce du cancer du sein au Maroc (GDPCS), l’existence d’un registre du cancer au Maroc), l’épidémiologie du cancer du sein (incidence et prévention du cancer du sein), les connaissances sur les facteurs de risque du cancer du sein, les connaissances sur les symptômes du cancer du sein, les connaissances sur le dépistage du cancer du sein par examen clinique des seins et mammographie (âge et périodicité du dépistage) et les sources d’information concernant le cancer du sein. La collecte des données a été faite par un questionnaire prétesté auto-administré. L’enquête s’est déroulée de Janvier 2015 à Avril 2015. Les réponses sur l’incidence ont été considérées correctes si elles étaient comprises entre 35 et 45 pour 100000 femmes selon le registre des cancers du Grand Casablanca. Pour les connaissances sur les limites d’âge inférieur et supérieur de l’ECS ainsi que sa périodicité on a utilisé les recommandations du guide de détection précoce du cancer du sein au Maroc: limite d’âge inférieur = 45 ans, limite d’âge supérieur = 70 ans, périodicité = 1fois/2ans. Concernant les connaissances sur les limites d’âge inférieur et supérieur de la mammographie ainsi que sa périodicité on a utilisé la combinaison des deux recommandations suivantes: l’US Preventive Services Task Force (USPSTF) qui recommande de faire un dépistage biannuel par mammographie chez les femmes entre 50 et 74 ans et l’American cancer society (ACS) qui recommande une mammographie annuelle chez toute femme au-dessus de l'âge de 40 ans.

**Analyse des données :** l’analyse des données a été faite grâce au Logiciel SPSS version 16.0 sous Windows. Pour les variables quantitatives on a calculé la moyenne et l’écart type, pour les données qualitatives: les proportions et les effectifs. Pour l’analyse bivariée on a utilisé le test de Khi 2 pour la comparaison des pourcentages et le test d’Anova à un facteur pour la comparaison des moyennes. Le seuil de signification a été fixé à 5%.

**Considérations éthiques :** l’anonymat et le respect de la confidentialité des données ont été assurés. Le consentement éclairé des MG a été obtenu oralement après les avoir informés des objectifs de l’enquête.

## Résultats

**Caractéristiques sociodémographiques et professionnelles des MG :** parmi les 97 MG ciblés par notre étude, 85 médecins ont répondu au questionnaire, soit un taux de participation de 87%. Le taux de participation était plus élevé dans le secteur public (89,5%) par rapport au secteur privé (85,7%). Le manque de temps était le motif de non-participation le plus évoqué. L’âge moyen des MG était de 49,6 (± 8,1 ans), 52,9 % étaient des femmes, 84,7% exerçaient en milieu urbain. La moyenne de l’ancienneté d’exercice était de 19,7 (± 8 ans).

**Outils stratégiques de contrôle du cancer du sein:** parmi les médecins interrogés, 77,6% connaissaient l’existence d’un PNPCC au Maroc, 62,4% connaissaient l’existence d’un guide de détection précoce du cancer du sein et 67,1% ont rapporté l’existence d’un registre du cancer au Maroc:l’analyse bi variée a montré que la connaissance des outils stratégiques était influencée par l’ancienneté d’exercice et le secteur d’activité: Les MG qui étaient informés de l’existence d’un guide de détection précoce du cancer du sein et d’un registre du cancer du sein étaient en moyenne moins anciens que les MG qui ne l’étaient pas, cette différence était statistiquement significative (p respectivement égale à 0,005 et 0.002) (Tableau 1). Une relation statiquement significative a été trouvée entre le secteur d’activité et les connaissances sur les outils stratégiques de contrôle et de dépistage du cancer du sein, où le nombre des médecins généralistes informés sur ces outils étaient plus élevé dans le secteur public que dans le secteur privé (Tableau 2). Dans ce travail, le genre ne semblait pas influencer les connaissances des MG concernant les outils stratégiques de contrôle et de dépistage du cancer du sein.

**Tableau 1:** Influence de l’ancienneté d’exercice sur les connaissances des MG de la préfecture de Mohammedia concernant les outils stratégiques de contrôle et de dépistage du cancer du sein, (n=85)

Connaissance / Ancienneté d’exercice	n	Moyenne± écart-type	P
**Cancer du sein est un problème majeur de santé publique au Maroc :**			
-Oui	75	19,45±7.99 ans	0.358
-Non	10	21.9 ±6.72 ans	
**Connaissances des MG sur l’existence du plan national de lutte et de contrôle du cancer**			
-Oui	66	19,24±8.08 ans	0.278
-Non	19	21.47±6.93 ans	
**Connaissances des MG sur l’existence d’un guide de détection précoce du cancer du sein**			
-Oui(43)	53	17.92±7.69 ans	**0.005**
-Non(42)	32	22.75±7.27 ans	
**Connaissances des MG sur l’existence d’un registre du cancer du sein**			
-Oui(43)	57	17.89±7.24 ans	**0.002**
Non(42)	28	23.50±7.84 ans	
**Connaissances des MG sur l’existence d’une circulaire ministérielle du cancer du sein :**			
-Oui(43)	55	18.56±7.58 ans	0.061
-Non(42)	30	1.90±8.01 ans	

**Tableau 2:** Influence du secteur d’activité sur les connaissances des MG de la préfecture de Mohammedia concernant les outils stratégiques de contrôle et de dépistage du cancer du sein (n = 85)

Connaissances/Secteur d’exercice	n	%	P
**Cancer du sein est un problème majeur de santé publique au Maroc :**			
-Public (43)	38	50.7	1
-Privé (42)	37	49.7	
**Connaissances des MG sur l’existence du plan national de lutte et de contrôle du cancer :**			
-public(43)	**39**	59.1	0.003
-Privé(42)	**27**	40.9	
**Connaissances des MG sur l’existence d’un guide de détection précoce du cancer du sein :**			
-Public(43)	**34**	64.2	0.001
-Privé(42)	19	35.8	
**Connaissances des MG sur l’existence d’un registre du cancer du sein :**			
-Public(43)	**39**	68.4	**<0.001**
-Privé(42)	18	31.6	
**Connaissances des MG sur l’existence d’unecirculaire ministérielle du cancer du sein :**			
-Public(43)	**39**	70.9	**<0.001**
-Privé(42)	**16**	29.1	

**Epidémiologie du cancer du sein:** parmi les 85 MG interrogés, 55 ont répondu à la question sur l’incidence du cancer du sein au Maroc. Parmi eux (80%) avaient donné une réponse incorrecte. Quatre-vingt-huit pour cent des MG ont reconnu le cancer du sein comme un problème majeur de santé publique au Maroc. L’analyse bi variée a montré que le nombre de MG (n=85) reconnaissant le cancer du sein comme un problème de santé publique était plus élevé dans le milieu urbain (89.3%) que dans le milieu rural (10.7%), cette différence était statistiquement significative (p=0,006).

**Facteurs de risque du cancer du sein:** le facteur de risque le plus mentionné était la présence d’ATCD familiaux de cancer du sein citée par 86,8 % des MG, vient en second lieu l’utilisation des contraceptifs par voie orale ou injectable rapportée par 56,6% MG. Les autres facteurs les plus souvent rapportés par les MG étaient l’âge avancé (34,2%), la nulliparité (32,9%), le tabac (22,4%), le traitement hormonal substitutif (11,8%). Les facteurs de risque les moins cités par les MG étaient, l’âge précoce des premières règles (≤12 ans) (10.5%), l’alimentation (10.5%), la ménopause tardive (9.2%).

**Symptômes du cancer du sein:** le symptôme le plus mentionné par les MG était la palpation d'un nodule du sein cité par 94% des MG suivi de la présence d’adénopathie (ADP) axillaire cité par 42.4% des MG. Un tiers des médecins interrogés ont identifié l’écoulement mamelonnaire et la rétraction mamelonnaire (respectivement 34.8% et 31.8%), et (27.4%) ont mentionné l’inflammation.

**Connaissances des MG sur le dépistage du cancer du sein:** pour l’autopalpation la majorité (95,3%) des MG étaient d’accord avec le fait qu’elle est importante pour le diagnostic précoce du cancer du sein et 88,2% étaient d’accord avec le fait qu’elle réduisait la mortalité par cancer du sein. Concernant l’examen clinique des seins, 98,8% des MG pensaient qu’il était important pour le diagnostic précoce du cancer du sein et 90,6 % pensaient que l’amélioration du pronostic était le but recherché. Quant à la mammographie, 91,8% des MG pensaient que l’amélioration du pronostic était sa principale finalité. Concernant l’âge de dépistage par Examen clinique des seins, la limite d’âge supérieure du dépistage a été reconnue par le tiers des médecins généralistes participants à l’étude (35.3%) alors que la limite d’âge inférieure a été reconnue par 17.6% des MG. L’intervalle de dépistage a été identifié par 23.5% des MG. Quant à l’âge de dépistage par mammographie, 64.7% des MG interrogés ont pu identifier la limite inférieure et seulement 3.5 % ont pu identifier la limite d’âge supérieure de dépistage. L’intervalle de dépistage a été reconnu par plus de la moitié des MG (55.3%). Par ailleurs l’analyse bi variée a retrouvé une association statistiquement significative entre les connaissances sur la limite d’âge inférieure du dépistage par ECS et le secteur d’activité. Les MG du secteur public sont plus informés que ceux du secteur privé avec un p=0,003 (Tableau 3). Concernant les connaissances sur la limite d’âge supérieure sur le dépistage par ECS, on a retrouvé une association statistiquement significative avec le genre (les MG femmes étaient plus informées que les MG hommes) et le secteur d’activité (les MG du secteur public étaient plus informés que les MG du secteur privé) avec (respectivement p= 0,024 et p<0,001) (Tableau 4). Pour les connaissances sur la limite d’âge inférieure sur le dépistage par mammographie, on a retrouvé une association significative avec le genre. Les MG femmes étaient plus informés que les MG hommes avec un p=0, 012. Dans notre étude aucune des caractéristiques sociodémographiques ou professionnelles ne semblaient influencer la connaissance de l’intervalle du dépistage par mammographie et ECS. La principale source d’information des MG sur le cancer du sein était la formation médicale initiale (63.5%), suivie des séminaires (52.9%). Les autres sources d’information étaient les congrès, internet, les collègues, la formation continue, les revues médicales rapportés respectivement par 23,5%, 14,1%, 10,6%, 9,4% et 1,2% des MG.

**Tableau 3:** Connaissances de la limite d’âge inférieure de dépistage par ECS du cancer du sein selon les caractéristiques des MG (n=85)

Limite d’âge inférieure de dépistage ECS/Caractéristiques MG	n	%	Moyenne ± écart type	p
**Genre**				
Masculin (40)	5	33,3	–	0,27
Féminin (45)	10	66,7	–	
**Milieu**				
Urbain (72)	13	86,7	–	1
Rural (13)	2	13,3	–	
**Secteur**				
Public (43)	13	86,7	–	**0,003**
Privé (42)	2	13,3	–	
**Ancienneté**				
Oui	15	–	17,87±5,8	0,311
Non	70	–	20,14±8,21	

**Tableau 4:** Connaissances de la limite d’âge supérieure de dépistage par ECS du cancer du sein selon les caractéristiques des MG (n=85)

Intervalle de dépistage mammographie/Caractéristiques MG	n	%	Moyenne ± écart type	p
**Genre**				
Masculin (40)	9	30	–	**0,024**
Féminin (45)	21	70	–	
**Milieu**				
Urbain (72)	26	86,7	–	1
Rural (13)	4	13,3	–	
**Secteur**				
Public (43)	24	80	–	**˂0,001**
Privé (42)	6	20	–	
**Ancienneté**				
Oui	30	–	18,10±7,45	0,156
Non	55	–	20,64±7,99	

## Discussion

Depuis 2010, au Maroc le dépistage du cancer du sein constitue une priorité en matière de lutte contre le cancer, un programme national de prévention et de contrôle du cancer a été mis en place et a retenu parmi ses priorités la détection précoce des cancers du sein et du col de l'utérus [6]. Notre étude était la première réalisée, selon nos connaissances au niveau de la préfecture de Mohammedia, elle était exhaustive incluant 97 MG public et privé avec un taux de réponse élevé de 87%. Mais comme pour toute étude déclarative, il peut y'avoir un écart entre ce que le médecin dit et ce qu'il fait réellement, cependant nos résultats rejoignent ceux d'autres études dans des contextes comparables ce qui permet de penser que nos résultats sont valides et interprétables. Presque un quart (22,3%) des MG ignoraient l'existence d'un PNPCC, le niveau de connaissance était plus défaillant dans une étude faite à Beni Mellal où 49.3% des MG participants à l'étude ne connaissaient pas son existence [17]. Plus du tiers (37.6%) des MG ne connaissaient pas l'existence d'un GDPCS au Maroc, un niveau de connaissance plus bas (71.8%) a été rapporté dans l'étude de Beni Mellal [17]. Trente-trois pour cent des MG n'avaient aucune information sur l'existence d'un registre du cancer au Maroc, le même constat a été retrouvé dans l'étude de Beni Mellal où 45% des MG n'étaient pas au courant de l'existence d'un registre du cancer au Maroc [17]. Ces différences peuvent être expliquées par le fait que la préfecture de Mohammedia fait partie des sites pilotes où ce programme de détection précoce du cancer du sein a été intégré alors qu'il n'était pas encore mis en place dans la province de Beni Mellal et la Wilaya de Fès. La majorité des MG (88,2%) considéraient le cancer du sein comme un problème de santé publique au Maroc. Ceci était compatible avec l´étude faite en Arabie Saoudite [18], l'étude faite au Nigeria [19] et l'étude faite à Fès [20], où respectivement 90%, 86.7% et 88.6% des Médecins considéraient le cancer du sein comme un problème de santé publique dans leurs pays. On a constaté qu'il y avait une tendance à la diminution des connaissances des médecins concernant ces outils avec l'augmentation de l'ancienneté d'exercice. Ceci peut être expliqué par le fait que les jeunes médecins avaient des connaissances plus actualisés par rapport aux médecins plus anciens. Les MG du secteur public étaient plus informés sur ces outils que les MG du secteur privé. Un effort de communication concernant ces outils stratégiques de lutte contre le cancer parait nécessaire pour augmenter l'adhérence et la participation des MG, et notamment ceux du secteur privé à tout programme du ministère de la santé notamment les actions de lutte contre le cancer. Le nombre de MG reconnaissant le cancer du sein comme un problème de santé publique était plus élevé dans le milieu urbain (89.3%) que dans le milieu rural (10.7%), cette différence était statistiquement significative (p=0,006). Ceci peut être expliqué par le fait que les MG exerçant dans le milieu urbain avaient plus accès à l'information notamment les actions du ministère de la santé par rapport au MG exerçant dans le milieu rural.

Par contre, nous n'avons pas trouvé d'association entre les connaissances des outils stratégiques de contrôle de cancer du sein et le genre des MG. Concernant l'épidémiologie du cancer du sein, on a constaté un manque de connaissance en effet 80% des 55 MG qui ont répondu à la question sur l'incidence ont donné une réponse incorrecte. Le même résultat a été rapporté dans une étude tunisienne où seulement 13.4% ont répondu correctement à la question [21]. Par contre dans une étude réalisée à Fès les MG avaient une meilleure connaissance où 50% d'entre eux avaient donné une réponse correcte [20]. Le facteur de risque le plus rapporté pas les MG était la présence d'ATCD familiaux de cancer du sein (86.8%). Ce chiffre est similaire à ceux constaté dans d'autres études faites au Canada (99 %) [22], au Pakistan (96 %) [23], en Arabie Saoudite (91.9%) [18] et à Fès (93.6%) [20]. L'utilisation des contraceptifs comme facteur de risque du cancer du sein a été identifiée par 56.6% des MG, des pourcentages plus élevées ont été rapportés par une étude réalisée à Rabat (78%) [8], l'étude réalisée à Fès (71.7%) [20], un pourcentage plus faible a été retrouvé dans une étude Angolaise (38%) [24]. L'âge avancé a été cité par 34.2% des MG, des résultats similaires ont été retrouvés dans une étude australienne qui a révélé que seulement 25% des médecins interrogés avaient reconnus l´âge avancé comme facteur de risque du cancer du sein [25] et l'étude de Rabat qui rapporte un taux de 47% [7]. Contrairement à l'étude réalisée en Arabie Saoudite où 82.1% des MG ont pu identifier l'âge avancé comme facteur de risque du cancer du sein [18], 75.9% rapporté dans l'étude de Fès [20]. Le niveau de connaissances des facteurs de risque de cancer du sein par les MG était insuffisant ceci a été également rapporté dans une étude iranienne où les connaissances des MG sur les facteurs de risque de cancer du sein n´étaient pas aussi satisfaisantes [26]. Concernant les symptômes du cancer du sein, le symptôme le plus mentionné par les MG était la présence d'un nodule du sein (93.3%), des pourcentages aussi élevés ont été retrouvé dans l'étude du Pakistan (95%) [23], et l'étude saoudienne (86.2%) [18]. Dans une autre étude réalisé en Ethiopie, ils ont trouvé un pourcentage plus faible (58.1%) [27]. En revanche, les autres symptômes ont été mentionnés par un nombre moins important de médecins. En effet, un tiers des médecins interrogés ont identifié l'écoulement mamelonnaire et la rétraction mamelonnaire (34.8 %, 31.8% respectivement), 42.4% ont mentionné la présence d'ADP axillaire, et seulement (27.4%) ont mentionné l'inflammation.

Ces pourcentages étaient faibles par rapport à l'étude saoudienne où le niveau de connaissance des médecins était plus élevé, l'écoulement mamelonnaire a été reconnu par 94.3% des médecins, la rétraction mamelonnaire par 90.2% des médecins, l'inflammation par 87% des médecins, et la présence d'ADP axillaire par 72% des médecins interrogés [18]. Dans l'étude éthiopienne, les pourcentages étaient de 51.1% pour l'écoulement mamelonnaire, 54.8 % pour la rétraction mamelonnaire et 54.1 % pour l'inflammation [27]. La différence retrouvée dans les résultats entre les différentes études et la nôtre peut être expliquée par le fait qu'il n'existe pas un instrument de collecte validé pour pouvoir comparer les différents travaux. On a utilisé dans notre questionnaire des questions ouvertes et des questions fermées. Dans les questions ouvertes les réponses peuvent être incomplètes car elles dépendent des acquis des MG, dans les questions fermées les réponses peuvent être surestimées car les réponses sont suggérées comme elles peuvent être limitées car il y a un choix limité de réponses. Les connaissances des MG sur l'âge à partir duquel l'examen clinique des seins devait être fait, indiquaient que seulement 17.6% des médecins rapportaient que les femmes devaient être dépistées à partir de 45 ans et (35.3%) des MG rapportaient que le dépistage devait être fait jusqu'un âge de 70 ans. Pour la périodicité de l'examen, chez 23.5% des MG l'examen clinique des seins devait être fait tous les deux ans. Le niveau de connaissances sur l'âge et les méthodes de dépistage par ECS était défaillant, ceci était rapporté également dans l'étude de Fès ou 24,8% des MG rapportaient que les femmes doivent être dépistées à partir de l'âge de 45 ans et 26.7% rapportaient que le dépistage doit être fait jusqu'à l'âge de 70 ans. Pour la périodicité de l'examen, 15.6% des MG rapportaient que l'examen clinique des seins doit être fait tous les deux ans [20]. Contrairement à l'étude Saoudienne ou le niveau de connaissance était meilleur avec 71% des médecins interrogés qui ont pu identifier l'âge et la périodicité de dépistage de cancer du sein par ECS [18]. Concernant le dépistage par mammographie, 64.7% des MG interrogés ont pu identifier la limite d'âge inférieure, alors que seulement 3.5% ont pu identifier la limite d'âge supérieure de dépistage. L'intervalle de dépistage a été reconnu par (55.3%) des MG. Même constat dans l'étude de Fès ou selon 37.6% des médecins participants à l'étude les femmes doivent être dépistées par mammographie à partir d'un âge supérieur à 45 ans et jusqu'à l'âge de 70 ans chez 26.2% des MG. Pour la périodicité de l'examen, plus de la moitié des MG (56,8%), rapportaient que la mammographie doit être faite tous les 2 ans [20]. Dans l'étude Saoudienne 84.6 % des médecins interrogés ont pu identifier l'âge et la périodicité du dépistage du cancer du sein par mammographie [18].

Une association statistiquement significative a été retrouvée entre les connaissances sur la limite d'âge inférieure du dépistage par ECS et le secteur d'activité. Les MG du secteur public étaient plus informés que ceux du secteur privé avec un p=0,003. Pour les connaissances sur la limite d'âge supérieure sur le dépistage par ECS, on a retrouvé une association statistiquement significative avec le genre et le secteur d'activité (respectivement p= 0,024 et p <0,001). Pour les connaissances sur la limite d'âge inférieure sur le dépistage par mammographie, on a retrouvé une association significative avec le genre. Les MG femmes étaient plus informés par rapport aux MG hommes avec un p=0, 012. Actuellement il n'y a pas encore de consensus international sur l'âge de début ni sur la périodicité du dépistage mais tous s'accordent que le choix doit prendre en compte les moyens de chaque pays. Par exemple aux états unis l'US Preventive Services Task Force (USPSTF) recommande de faire un dépistage biannuel par mammographie chez les femmes entre 50 et 74 ans [28]. En France le programme national de dépistage organisé du cancer du sein repose sur une mammographie bilatérale tous les deux ans aux femmes âgées de 50 jusqu'à 74 ans à l'exclusion de celles présentant un risque aggravé de cancer du sein [29]. Au Maroc, un grand effort a été fourni pour mettre en place un système organisé de dépistage, de diagnostic précoce et de traitement du cancer du sein dans le cadre du PNPCC. Dans le guide de détection précoce du cancer du sein, la population cible du dépistage sont les femmes âgées de 45 ans à 69 ans révolus et les femmes ayant un antécédent familial de cancer du sein (grand-mère, mère, tante, sœur). Le test de dépistage retenu par le PNPCC est l'examen clinique des seins, ce test doit être refait tous les deux ans si le résultat du test antérieur est négatif [6]. La majorité des médecins interrogées (63.5%) avaient obtenu leurs informations et connaissances pour le cancer du sein via la formation médicale initiale. Même constat dans une étude faite en Ethiopie [27] et à Singapour [30], où la principale source d'information était également la formation médicale initiale avec des pourcentages plus élevés (respectivement 78.9%, 74%). Ce travail a montré que les MG n'étaient pas en majorité suffisamment informés sur le cancer du sein et sur ses outils stratégiques de contrôle et de dépistage.

## Conclusion

Les résultats de ce travail montrent qu’il existe une défaillance concernant les connaissances des médecins généralistes en matière de détection précoce du cancer du sein dans la préfecture de Mohammedia où le programme de détection précoce du cancer du sein est déjà opérationnel. Il est nécessaire de renforcer les connaissances et les pratiques des médecins généralistes par la promotion de la formation initiale et continue dans le domaine de la détection précoce du cancer du sein. Il serait également souhaitable de renforcer les actions de sensibilisation des femmes par rapport au dépistage du cancer du sein pour obtenir une meilleure adhésion à ces programmes.

### Etat des connaissances actuelle sur le sujet

- La plupart des études faites dans ce sens étaient comparatives entre les médecins généralistes et les infirmiers ou les étudiants en médecines;
- Le résultat tendait toujours vers une connaissance des médecins généralistes qui était plus pertinente par rapport aux autres participants;
- D’après la bibliographie on a constaté une défaillance au niveau des connaissances concernant le cancer du sein et son dépistage concernant les études marocaines (Rabat, Fès) par rapport aux études occidentales.

### Contribution de notre étude à la connaissance

- Les médecins généralistes représentent en général le premier contact avec les patients, donc en matière de dépistage ils sont les leaders, c’est pour cela qu’on s’est focalisé dans notre travail sur cette population des médecins généralistes et notre étude étaient exhaustive en incluant les médecins du secteur privé et public;
- Notre étude vient confirmer les résultats retrouvés dans les études similaires dans le Maroc, elle fait également ressortir les facteurs associés (ancienneté, le sexe, le secteur d’activité, le milieu du travail) à cette défaillance au niveau des connaissances « même si ça reste une étude descriptive » on a pu émettre des hypothèses pour expliquer ce manque de connaissances. Il faudra bien sûr compléter avec des études analytiques pour confirmer ces hypothèses;
- Notre étude a mis également le point sur la défaillance de l’application du programme national de détection précoce du cancer du sein dans cette préfecture (Mohammedia) qui normalement devait être opérationnel vu qu’il est déjà instauré depuis longtemps.
